# Identification of Human Enzymes Using Amino Acid Composition and the Composition of *k*-Spaced Amino Acid Pairs

**DOI:** 10.1155/2020/9235920

**Published:** 2020-05-22

**Authors:** Lifu Zhang, Benzhi Dong, Zhixia Teng, Ying Zhang, Liran Juan

**Affiliations:** ^1^School of Aeronautics and Astronautics, University of Electronic Science and Technology of China, Chengdu, China; ^2^Institute of Fundamental and Frontier Sciences, University of Electronic Science and Technology of China, Chengdu, China; ^3^Information and Computer Engineering College, Northeast Forestry University, Harbin, China; ^4^Department of Pharmacy, Heilongjiang Province Land Reclamation Headquarters General Hospital, Harbin, China; ^5^School of Life Science and Technology, Harbin Institute of Technology, Harbin, China

## Abstract

Enzymes are proteins that can efficiently catalyze specific biochemical reactions, and they are widely present in the human body. Developing an efficient method to identify human enzymes is vital to select enzymes from the vast number of human proteins and to investigate their functions. Nevertheless, only a limited amount of research has been conducted on the classification of human enzymes and nonenzymes. In this work, we developed a support vector machine- (SVM-) based predictor to classify human enzymes using the amino acid composition (AAC), the composition of *k*-spaced amino acid pairs (CKSAAP), and selected informative amino acid pairs through the use of a feature selection technique. A training dataset including 1117 human enzymes and 2099 nonenzymes and a test dataset including 684 human enzymes and 1270 nonenzymes were constructed to train and test the proposed model. The results of jackknife cross-validation showed that the overall accuracy was 76.46% for the training set and 76.21% for the test set, which are higher than the 72.6% achieved in previous research. Furthermore, various feature extraction methods and mainstream classifiers were compared in this task, and informative feature parameters of *k*-spaced amino acid pairs were selected and compared. The results suggest that our classifier can be used in human enzyme identification effectively and efficiently and can help to understand their functions and develop new drugs.

## 1. Introduction

Enzymes, also known as biocatalysts, are proteins that can catalyze chemical reactions in living cells efficiently and specifically, and they play a key role in the survival of humans, other animals, and plants. Over the last few decades, enzymes in increasing numbers have been identified and have been found to have a variety of properties and play diverse roles in the survival, growth, and development of organisms.

Depending on the properties of the reaction catalyzed, enzymes are classified into six classes according to enzyme commission (EC) numbers [1]: oxidoreductases, transferases, hydrolases, lyases, isomerases, and ligases. Owing to the specificity of enzymes, i.e., an enzyme can only catalyze a specific chemical reaction in a cell, accurately classifying and predicting enzyme classes is of vital importance when searching for unknown enzymes and developing new drugs, including zymin.

The traditional approach to the identification of proteins through wet experimental methods has typically been time and resource intensive. With the development of protein sequencing technology and improvements in computing power, computational methods based on amino acid sequence data of peptides, especially machine learning methods, have been widely used to classify and predict the function of diverse classes of proteins [2–7].

Currently, several researchers have focused on developing methods that can be used for the identification of enzymes. Jensen et al. first predicted enzyme classes using sequence-based physicochemical features and an Artificial Neural Network (ANN) in 2002 [8]. Chou and Cai proposed the GO-PseAAC predictor, which combined gene ontology (GO) and Pseudo amino acid composition (PseAAC) as features to search for and used the nearest neighbor algorithm approach [9]. Later, Cai et al. first applied the SVM algorithm to enzyme classification [10] and combined functional domain composition (FunD) with PseAAC to predict the classes of enzymes [11, 12]. Furthermore, a predictor named EzyPred was developed by Shen and Chou that uses FunD and the Pseudo position-specific scoring matrix (PsePSSM) as features [13]. In 2009, Nasibov and Kandemir-Cavas classified enzymes by the *K*-nearest neighbor (KNN) method and the minimum distance-based predictor using AAC [14]. Concu et al. provided a distinctive method using the 3D structure rather than sequence information [15]. Qiu et al. developed a method based on PseAAC and discrete wavelet transform (WT) that was trained by the SVM algorithm [16]. Shi and Hu used low-frequency power spectral density and increment of diversity, combined with AAC and PseAAC, and built an SVM-based predictor [17]. In addition, Zou et al. introduced a multilabel learning method to identify multifunctional enzymes [18]. Later, a new method was put forward by Niu et al. that used a protein-protein network [19]. In recent years, deep-learning methods like convolutional neural networks were used for the classification of enzymes and achieved good results [20, 21].

All of these classification methods improved the classification performance based on previous research. Nevertheless, all of these researchers concentrated on classifying different types of enzymes, and very few methods have been developed to predict whether a protein is an enzyme or a nonenzyme. Wu et al. devoted themselves to this issue and designed an SVM-based method combining PseAAC with the rigidity [22], flexibility, and irreplaceability of amino acids to identify human enzyme classes. However, this method only reached an overall accuracy of 72.6% by 5-fold cross-validation using 372 features, and thus, the performance of this task needs to be further improved.

On the basis of the above research, in this work, we developed a new machine learning method to classify human enzymes and nonenzymes. First, we introduced a feature representation strategy based on AAC and the composition of *k*-spaced amino acid pairs (CKSAAP). Next, for features represented by the methods above, the feature selection technique based on analyses of variance (ANOVA) was applied to minimize the features we used and to improve its overall accuracy. Finally, the selected features were fed into the classifiers found from SVM for training. As a result, an accuracy of 76.46% and 76.21% by 6-fold cross-validation was achieved in the training set and test set, respectively, by using 40 feature parameters. Furthermore, the performances of different feature representation strategies under the SVM classifier and the performances of different classifiers were compared and discussed, and important feature parameters in this task were selected and compared.

## 2. Materials and Methods

### 2.1. Datasets

The training sequence data used in this study were first reported by Wu et al. [22] and were obtained from the Universal Protein Resource (UniProt), the protein database with the most abundant information and resources; the training sequence data were composed of data from three databases: Swiss-Prot, TrEMBL, and PIR-PSD [23]. Six subclasses of human enzymes and nonhuman enzymes can be filtered and downloaded for free. To ensure the correctness and representativeness of the training data, the following data preprocessing process was used: (1) Human enzyme sequences of enzymes whose function had not been experimentally verified and those labeled as fragments were eliminated. (2) Enzyme sequences containing ambiguous residues (“B,” “J,” “O,” “U,” “X,” and “Z”) were excluded. (3) The CD-HIT program was applied to remove highly similar enzyme sequences using 30% as the cutoff of sequence identity [24, 25].

**Figure 1:**
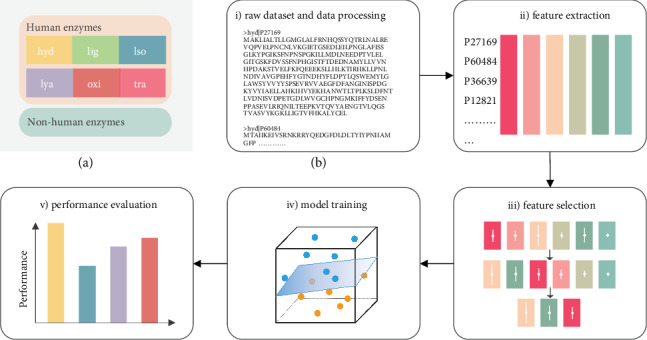
Overall workflow. (a) The original sequence dataset used. The dataset consists of human enzymes and nonhuman enzymes. Among them, human enzymes consist of 6 subsets, which represent the catalytic effects on different types of biochemical reactions: oxidoreductases, transferases, hydrolases, lyases, isomerases, and ligases. (b) The workflow of our study. Raw protein sequences were first preprocessed and fed into a feature extraction process, and then, a three-step feature selection technique was used to reduce feature parameters. Last, the selected feature parameters were used to train an SVM-based model, and the performance of the model was evaluated by several evaluation indexes.

After the above data preprocessing steps were completed, 1117 human enzymes and 2099 nonhuman enzymes were selected as training sequences in the analysis. Among them, the human enzyme sequences consist of 6 subclasses, as shown in Figure 1(a), with the overall workflow in our study shown in Figure 1(b).

Furthermore, to evaluate the effect of the model more accurately, a set of test data was selected from the dataset used by Cai and Chou [11] and downloaded from UniProt [23]; these data included a total of 1954 sequences, including 684 enzymes and 1270 nonhuman enzyme sequences, respectively.

### 2.2. Feature Extraction

One of the most important steps in our method was to extract the feature vector of the selected sequences. Many works have focused on feature extraction of proteins. AAC [26, 27], dipeptide composition (DPC) [28, 29], Geary correction [30], composition-transition-distribution [10, 31, 32], PseAAC [33–37], and other feature extraction methods [38–40] have been proposed and widely applied to describe different kinds of protein primary sequences. Here, we presented and then applied AAC and CKSAAP to extract features.

The AAC encoding strategy calculates the frequency of each type of the 20 amino acids in a primary protein sequence [26], which can be formulated as follows:

(1) $$\begin{array}{c} \left\{\begin{array}{c} R_{\text{AAC}}={\left[\text{f}\left(1\right)\text{f}\left(2\right)\cdots \text{f}\left(i\right)\cdots \text{f}\left(20\right)\right]}_{20}^{T},\\ f\left(i\right)=\frac{N\left(i\right)}{L}\left(1\leq i\leq 20\right), \end{array}\right. \end{array}$$

where *N*(*i*) denotes the number of the amino acid types *i* (i.e., A, C, D, E, etc.) and *L* denotes the length of the sequence. This strategy obtains a 20-D feature vector for each primary sequence.

The CKSAAP encoding strategy reflects the short-range interaction of the sequence. The frequency of 400 amino acid pairs in *k*-space is calculated using this strategy [41]. The frequency can be defined as follows:

(2) $$\begin{array}{c} \left\{\begin{array}{c} R_{\text{CKSAAP}}={\left[f\left(1,1\right)\;f\left(1,2\right)\cdots f\left(i,j\right)\cdots f\left(20,20\right)\right]}_{400}^{T},\\ f\left(i,j\right)=\frac{N\left(i,j\right)}{L-k}\left(1\leq i,j\leq 20\right), \end{array}\right. \end{array}$$

where *N*(*i*, *j*) denotes the number of the amino acid types *i* and *j* in *k*-space. *L* denotes the length of the sequence. This strategy obtains a 400-D feature vector for each primary sequence. Taking *k* = 1 as an example, there are 400 amino acid pairs in 1-space, i.e., A^∗^A, A^∗^C, A^∗^D, etc., where ^∗^ denotes other amino acids as the gap [42]. In this research, *k* = 0, 1, 2, 3, 4, and 5 are used to extract features and measure the comparative effectiveness. Therefore, the dipeptide composition (DPC) is the same descriptor as CKSAAP when *k* = 0 [43]. Moreover, in our work, features of sequences are extracted by the iFeature toolkit [44].

### 2.3. Feature Selection

Feature selection was utilized to optimize the prediction model and improve the accuracy of the human-enzyme classification task. In previous research, principal component analysis (PCA), the minimal redundancy maximal relevance (mRMR) algorithm [45, 46], the maximum relevance maximum distance (MRMD) algorithm [47], the genetic algorithm, etc., were proposed for feature selection and applied in protein classification. Here, ANOVA is used to select the most representative features.

ANOVA is an effective method used in statistics to test for a significant relationship between the selected variable and group variables [48, 49]. In our paper, ANOVA can be applied to measure the correlation between a selected feature and all features. The *F* statistic (*F*(*δ*)) of a feature *δ* is defined as follows:

(3) $$\begin{array}{c} F\left(\delta \right)=\frac{s_{\text{MSB}}^{2}\left(\delta \right)}{s_{\text{MSW}}^{2}\left(\delta \right)}, \end{array}$$

where *s*_MSB_^2^(*δ*) and *s*_MSW_^2^(*δ*) represent the mean square between (MSB) and the mean square within (MSW), respectively, which can be interpreted as the sample variance between groups and the sample variance within groups. In the theory of statistics, *F*(*δ*) satisfies the *F*-distribution, which is used for the significance test. However, in our study, we only focused on the relative values of *F*(*δ*) to indicate the correlation between the feature and the overall size. Features with a larger *F*(*δ*) are selected because a larger *F*(*δ*) implies that they are more strongly related to the group features and more likely to contribute to the classification.

### 2.4. Support Vector Machine

The SVM algorithm is one of the most popular machine learning algorithms which has been successfully applied in many areas [50–58]. The SVM algorithm is based on statistical learning theory and is widely used in various domains. In the field of protein prediction, SVM has been applied to predicting protein category, secondary structure, physical and chemical properties, etc. and has achieved remarkable results [31, 59–63].

The core idea of SVM is to map the vectors from a low-dimension input space to a high-dimension Hilbert space, in which a linear separating hyperplane is constructed by a kernel function, and to try to maximize the margin among the support vectors of each class by adjusting the linear separating hyperplane. Usually, varieties of kernel functions can be used in SVM algorithms, including linear function, polynomial function, sigmoid function, and radial basis function (RBF). Previous research has shown that RBF performs much better than the other three kinds of kernel functions. Hence, RBF was used in our work as the kernel function [31, 59–63].

During the course of algorithm implementation, the open-source package libSVM supplied by Chang and Lin was used to implement the SVM algorithm [64]. Two parameters, *c* and *γ*, related to loss function and kernel function, respectively, were optimized by the method of gridding search using 6-fold cross-validation.

### 2.5. Performance Evaluation

Overfitting is an inevitable problem in machine learning. To reduce the influence of overfitting on model training, jackknife cross-validation or *n*-fold cross-validation is used to examine the power of the model on the training set [65]. The jackknife cross-validation method divides the training set into *k* subsets randomly, one of which is used to verify the accuracy of the model, and the other *k*‐1 subsets are used to train the model. This method can avoid overfitting by generalizing the model with *k*-times repetition and is widely used in the machine learning process of small sample size data.

The performance of each model can be measured in terms of accuracy (ACC), sensitivity (SE), and specificity (SP) [66–72]. A confusion matrix can be set up with the help of the classification results, which further classifies the classification results of a binary classifier into four categories: true positive (TP), true negative (TN), false positive (FP), and false negative (FN) [73, 74]. These metrics are usually adopted to evaluate prediction quality [75–89]. Based on this, the parameters above can be expressed as follows:

(4) $$\begin{array}{c} \left\{\begin{array}{c} \text{ACC}=\frac{\text{TP}+\text{TN}}{\text{TP}+\text{TN}+\text{FP}+\text{FN}}*100\%,\\ \text{SP}=\frac{\text{TP}}{\text{TP}+\text{FN}}*100\%,\\ \text{SE}=\frac{\text{TN}}{\text{TN}+\text{FP}}*100\%, \end{array}\right. \end{array}$$

where ACC is used to evaluate the overall performance of the model and SE and SP are used to measure the predictive ability of the model for positive and negative cases. Higher values of these parameters represent a better prediction performance of the model.

In addition, the receiver operating characteristic (ROC) curve is applied to evaluate the performance of the model further [90–100]. ROC curves are used to illustrate the diagnostic ability of a binary classifier, which shows the changes of SP and SE with varied thresholds. The area under the ROC curve (AUC) can be used to determine which classifier performs better in a quantitative way. ROC curve analysis can reflect the real performance of the model, especially for an unbalanced dataset.

## 3. Results and Discussion

### 3.1. Comparison of Feature Extraction Methods

We first compared the performance of common feature extraction methods on the training set identified by the SVM classifier. Feature vectors with high dimensions were selected by ANOVA or mRMR methods, depending on which method could maximize accuracy. The features of the sequences were extracted by the iFeature toolkit [44] and were then selected and classified using MATLAB and libSVM. The accuracies of the various methods are shown in Supplementary Materials (available here), calculated by 6-fold cross-validation. We found that AAC and composition, transition, and distribution (CTD) descriptors can classify human enzymes accurately, with an accuracy from 74.4% to 75.9%, and that AAC can achieve the highest accuracy, which means the frequency of all 20 amino acids can provide the most useful information about human enzyme classification, and thus, more useful information can be added to AAC to improve the model's prediction performance.

Based on the above discussion, other descriptors can be added to AAC to improve the model. The results of the predicted accuracy using different added descriptors are shown in Table 1, where the feature selection technique in ANOVA and mRMR with higher accuracy was used. The control variable method is used to find the optimal feature extraction method. Specifically, the dimension used for feature selection is unchanged (30-D), and the performance of the SVM classifier under different feature extraction methods is compared to find the best feature extraction method for the identification of human enzymes. Based on the performance of the different descriptors on the training set, CKSAAP, which included not only information about the composition and sequence order but also information about the residue correlation, was determined to be the descriptor that can provide new valid information on the basis of AAC to improve the model performance.

### 3.2. Necessity of Feature Selection

**Figure 2:**
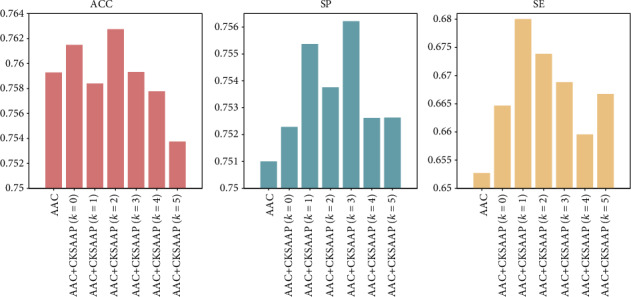
Comparison of SVM models trained by AAC alone versus AAC plus 6 types of CKSAAP.

Then, the performance of our method, using the AAC and CKSAAP descriptors as features, was measured in different dimensions that were selected to determine whether the feature selection method should be used to reduce redundant information and further improve the performance of our model. We employed AAC alone and AAC and 6 types of CKSAAP together as the predictor to train the SVM model. The results are presented in Figure 2. Relative to SE, SP, the ACC model using all of the features of AAC and CKSAAP was not much improved compared to using AAC alone and was even decreased, in spite of features in CKSAAP that include useless information that influences the precision of our model. This result could lead to the conclusion that a feature selection technique is necessary to reduce redundant information and improve the precision of our model.

### 3.3. Selection of Significant Features

After determining the feature selection techniques necessary to improve the prediction accuracy of the model, the size of the significant features of the CKSAAP descriptors that we selected needed to be identified. We used ANOVA to select informative *k*-spaced amino acid pairs. The definite means are as follows: (1) Evaluate all of the amino acid pairs and sort them according to the difference between the two types of amino acids. (2) Each CKSAAP feature is sequentially added to the parameter subset with AAC according to the sorted order. (3) The SVM-based model is trained using the parameter subset. Then, all of the results are compared to find the best feature subset of the significant features we selected.

**Figure 3:**
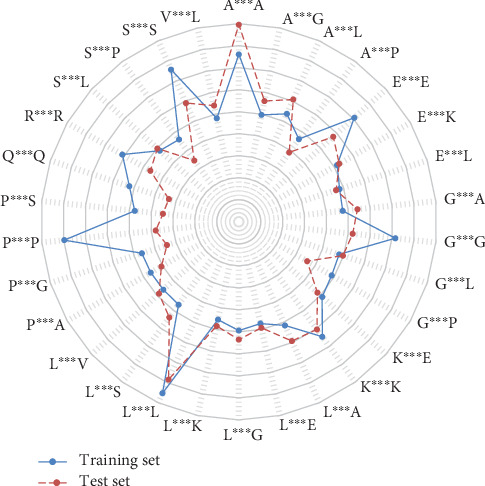
Results of the top 30 feature parameters of CKSAAP (*k* = 3). The radius of each point indicates the variance of the feature parameter in the training set or test set.

According to these methods, taking *k* = 3 as an example, the top 30 feature parameters of CKSAAP were selected and are shown as Figure 3, and the variance of 50 feature parameters in both the training and test sets are also shown. A^∗∗∗^A and L^∗∗∗^L have a large variance in both the training and test sets, foreshadowing that they contain more information.

**Figure 4:**
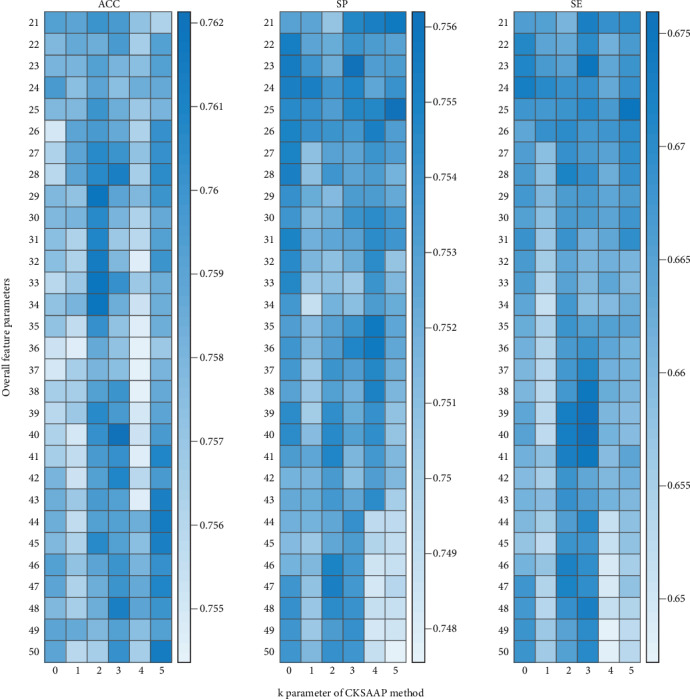
Results of ACC, SP, and SE of the model trained by 20 AAC parameters and 1–30 important CKSAAP parameters selected by the ANOVA technique.

We used the top 30 feature parameters of CKSAAP from ANOVA added into the AAC parameters to train the model, change the value of *k* during feature extraction, and change the number of features added to AAC at the same time to select the model with the best performance, instead of only changing the feature extraction method, and the results are shown in Figure 4. We obtained a maximum accuracy when we used 20 AAC parameters and 20 CKSAAP parameters (*k* = 3) for 40 feature parameters overall. The *c*/*γ* values used in the SVM-based model are 1.1487 (2^0.2^) and 147.0334 (2^7.2^), respectively. The accuracy reached 76.2135%, and SP and SE reached 0.7530 and 0.6760, respectively, which are all higher than the accuracy achieved in past research. We also measured the performance of the above model by making predictions on the test set and obtained an overall accuracy of 76.4585%, which indicates that the SVM model we established performs well in the classification of human enzymes. The 20 informative 3-spaced amino acid pairs that are used in the model training stage are L^∗∗∗^L, P^∗∗∗^P, A^∗∗∗^A, S^∗∗∗^S, G^∗∗∗^G, E^∗∗∗^E, K^∗∗∗^K, R^∗∗∗^R, A^∗∗∗^L, Q^∗∗∗^Q, E^∗∗∗^K, L^∗∗∗^A, K^∗∗∗^E, A^∗∗∗^G, L^∗∗∗^G, G^∗∗∗^P, S^∗∗∗^L, E^∗∗∗^L, V^∗∗∗^L, and G^∗∗∗^L (^∗^ indicates the other characters between two amino acids, i.e., the space), which may play important roles in human enzymes.

**Figure 5:**
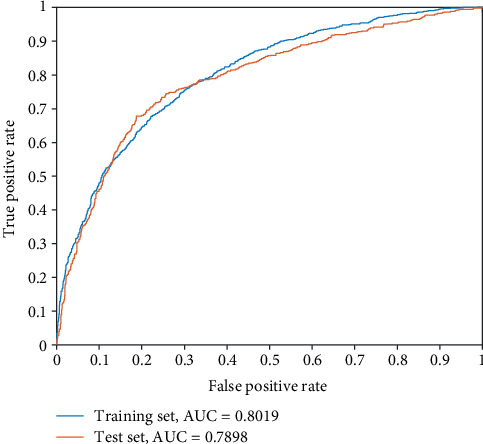
The ROC curves of our model on both the training set and test set, with AUCs of 0.8019 and 0.7898, respectively.

Furthermore, various mainstream classifiers, i.e., Naive Bayes, Random Forest, Logistic, *K*-nearest neighbor (KNN), and Ensembles for Boosting [102–105] are compared with our model in both the training set and the test set using 6-fold cross-validation in Table 2, and the result shows that the SVM-based classifier in our paper performs best. In addition, the ROC curve of our model performed well on both the training set and the test set, as shown in Figure 5, which confirms the classification effect of the model. The AUC reached 0.8019 and 0.7898 in the training set and the test set, respectively, demonstrating that our method for human-enzyme classification is effective and that more accurate classification results can now be obtained.

## 4. Conclusion

In this study, we proposed an effective and novel method to identify human enzymes using AAC and CKSAAP that is based on short-range interactions of amino acid pairs rather than the physicochemical properties of the sequences. By using ANOVA to select informative feature parameters, 20 amino acid pairs in 3-space are selected to add 20 residues and feed their frequency into an SVM classifier. The jackknife cross-validated accuracy was 76.46% in the training set, demonstrating that fewer feature parameters were used and a higher accuracy was reached compared to previous research. Moreover, we compared the performance of the model using different feature extraction methods, and the results showed that residue-frequency-based methods perform better than other methods, and a web server based on our method will be implemented in the future. In addition, some important feature parameters selected by ANOVA, e.g., A^∗∗∗^A and L^∗∗∗^L, may contain vital information in regard to the identification of human enzymes, which we hope to discuss more deeply in the future.

## Figures and Tables

**Table 1:** Accuracy of models trained with various feature parameters added into AAC by 6-fold cross-validation.

Feature parameters added into AAC	Feature selection method	Added number of features/total number of features	Accuracy
CTD-C [10]	mRMR	20/39	75.1547%
CTriad [101]	mRMR	30/343	71.0349%
DPC [28]	ANOVA	30/400	75.5569%
DDE [28]	ANOVA	30/400	67.0483%
TPC [26]	ANOVA	30/8000	75.5569%
PseAAC [33]	ANOVA	30/50	73.5075%
Geary [30]	mRMR	30/240	75.8706%
CKSAAP (*k* = 0~5)	ANOVA	30/2400	75.9282%
CKSAAP (*k* = 0)	ANOVA	30/400	75.7776%
CKSAAP (*k* = 1)	ANOVA	30/400	76.0885%
CKSAAP (*k* = 2)	ANOVA	30/400	75.7147%
CKSAAP (*k* = 3)	ANOVA	30/400	76.0878%
CKSAAP (*k* = 4)	ANOVA	30/400	75.8708%
CKSAAP (*k* = 5)	ANOVA	30/400	75.8701%

**Table 2:** Comparison of the performance of various mainstream classifiers and the classifier implemented in our paper. ACC, SP, and SE of different classifiers on both the training set and the test set are compared.

Classifiers	Training set			Test set		
	ACC	SP	SE	ACC	SP	SE
This work (SVM)	76.2135%	0.753	0.676	76.4585%	0.762	0.657
Naive Bayes	61.0697%	0.466	0.833	65.7625%	0.507	0.794
Random Forest	74.3781%	0.703	0.454	74.7691%	0.710	0.472
Logistic	69.5274%	0.598	0.374	68.4237%	0.587	0.329
KNN	62.8420%	0.474	0.646	63.0502%	0.480	0.658
Ensembles for Boosting	69.6206%	0.588	0.420	68.6796%	0.573	0.411

## Data Availability

In our experiment, the sequence data of the training set and the feature vectors of both the training set and the test set extracted by the iFeature toolkit are available online at https://github.com/Fu-Zhang/Identification-of-human-enzymes. The sequence data of the test set are available in the Supplementary Materials of Reference [11].

## Supplementary Materials

Supplementary Materials Accuracy of model training with various feature extraction methods by 6-fold cross-validation. Two feature selection methods, ANOVA and mRMR, are used and the feature selection method with higher accuracy is selected and included in the table.
