# Supplementary material for: Identification of Human Enzymes Using Amino Acid Composition and the Composition of k-Spaced Amino Acid Pairs
Source: Biomed Res Int. 2020 May 22;2020:9235920. doi: 10.1155/2020/9235920 (PMC7273372; doi:10.1155/2020/9235920)
Supplement: Supplementary Materials — Accuracy of model training with various feature extraction methods by 6-fold cross-validation. Two feature selection methods, ANOVA and mRMR, are used and the feature selection method with higher accuracy is selected and included in the table. [file 9235920.f1.docx]

**Supplementary Materials**

**Supplementary Material A** Accuracy of model training with various feature extraction methods by 6-fold cross-validation. Two feature selection methods ANOVA and mRMR are used and the feature selection method with higher accuracy is selected and included in the table.

| **Feature extraction method** | **Feature selection method** | **The selected number of features / Total number of features** | **Accuracy** |
| --- | --- | --- | --- |
| AAC[24] | / | 20/20 | 75.9282% |
| CKSAAP (k=0~5) | ANOVA | 50/2400 | 71.5037% |
| DDE[26] | ANOVA | 50/400 | 66.5842% |
| CTD-C[10] | mRMR | 22/39 | 74.5666% |
| CTD-T[10] | mRMR | 30/39 | 74.4740% |
| CTriad[99] | mRMR | 50/343 | 68.8428% |
| DPC[26]  (CKSAAP k=0) | ANOVA | 30/400 | 68.8122% |
| CKSAAP k=1 | ANOVA | 30/400 | 71.2065% |
| CKSAAP k=2 | ANOVA | 30/400 | 70.1493% |
| CKSAAP k=3 | ANOVA | 30/400 | 69.4652% |
| CKSAAP k=4 | ANOVA | 30/400 | 69.0299% |
| CKSAAP k=5 | ANOVA | 30/400 | 69.6828% |
| Moran[100] | ANOVA | 50/240 | 71.4552% |
| PseAAC[31] | ANOVA | 30/50 | 73.5075% |
| Geary[28] | mRMR | 50/240 | 71.1754% |
| KSCTriad[99] | mRMR | 30/343 | 68.8428% |
| TPC[24] | ANOVA | 50/8000 | 65.4394% |
